# The Effects of Socioeconomic Status, Clinical Factors, and Genetic Ancestry on Pulmonary Tuberculosis Disease in Northeastern Mexico

**DOI:** 10.1371/journal.pone.0094303

**Published:** 2014-04-11

**Authors:** Bonnie N. Young, Adrian Rendón, Adrian Rosas-Taraco, Jack Baker, Meghan Healy, Jessica M. Gross, Jeffrey Long, Marcos Burgos, Keith L. Hunley

**Affiliations:** 1 University of New Mexico, Department of Anthropology, Albuquerque, New Mexico, United States of America; 2 Universidad Autónoma de Nuevo León, Hospital José E. González, Tuberculosis Clinic, Monterrey, Nuevo León, Mexico; 3 Universidad Autónoma de Nuevo León, School of Medicine, Department of Immunology, Monterrey, Nuevo León, Mexico; 4 University of New Mexico, Geospatial and Population Studies, Albuquerque, New Mexico, United States of America; 5 University of New Mexico, Department of Internal Medicine, Albuquerque, New Mexico, United States of America; St. Petersburg Pasteur Institute, Russian Federation

## Abstract

Diverse socioeconomic and clinical factors influence susceptibility to tuberculosis (TB) disease in Mexico. The role of genetic factors, particularly those that differ between the parental groups that admixed in Mexico, is unclear. The objectives of this study are to identify the socioeconomic and clinical predictors of the transition from latent TB infection (LTBI) to pulmonary TB disease in an urban population in northeastern Mexico, and to examine whether genetic ancestry plays an independent role in this transition. We recruited 97 pulmonary TB disease patients and 97 LTBI individuals from a public hospital in Monterrey, Nuevo León. Socioeconomic and clinical variables were collected from interviews and medical records, and genetic ancestry was estimated for a subset of 142 study participants from 291,917 single nucleotide polymorphisms (SNPs). We examined crude associations between the variables and TB disease status. Significant predictors from crude association tests were analyzed using multivariable logistic regression. We also compared genetic ancestry between LTBI individuals and TB disease patients at 1,314 SNPs in 273 genes from the TB biosystem in the NCBI BioSystems database. In crude association tests, 12 socioeconomic and clinical variables were associated with TB disease. Multivariable logistic regression analyses indicated that marital status, diabetes, and smoking were independently associated with TB status. Genetic ancestry was not associated with TB disease in either crude or multivariable analyses. Separate analyses showed that LTBI individuals recruited from hospital staff had significantly higher European genetic ancestry than LTBI individuals recruited from the clinics and waiting rooms. Genetic ancestry differed between individuals with LTBI and TB disease at SNPs located in two genes in the TB biosystem. These results indicate that Monterrey may be structured with respect to genetic ancestry, and that genetic differences in TB susceptibility in parental populations may contribute to variation in disease susceptibility in the region.

## Introduction

Approximately one third of the world's population is infected with TB [1]. Among the majority of infected people, the immune response effectively neutralizes the bacteria in the lungs; these individuals are asymptomatic and not contagious [2]. Approximately 10% of infected individuals progress from latent TB infection (LTBI) to disease status, and, in the absence of treatment, about half of these individuals die.

Most TB-related deaths occur in low-income countries in Africa, Asia, and the Americas [1]. Rates of TB disease and death are comparatively lower in Western Europe and in people of Western European ancestry in the Americas and other regions. U.S. whites, for example, experience lower rates of TB disease than other national, racial, and ethnic groups. This disparity is largely attributed to socioeconomic factors associated with place of birth, income, education, and health care access [3]–[7]. Related factors that predispose individuals to exposure to TB and risk of developing disease include crowding, poor nutrition, and disease co-morbidities [8]–[10]. Genetic factors are also associated with TB susceptibility worldwide [11]–[15], although the role of regional differences in genetic susceptibility is less clear [16]–[18]. The objectives of this study are to identify the socioeconomic and clinical predictors of the transition from LTBI to pulmonary TB disease in the urban setting of Monterrey in northeastern Mexico, and to examine whether genetic ancestry plays an independent role in this transition.

The Monterrey Metropolitan Area (MMA) is the urban and industrial hub of Nuevo León. Rates of TB disease are high in the MMA compared to other regions of North America, and even other regions of Mexico [19]. The current rate of 16 new TB cases per 100,000 [19], [20] contrasts with a rate of 3.4 cases in the United States [21], and 5.8 in U.S. Hispanics [21]. Despite the fact that the MMA is one of the wealthiest areas in Mexico, the state of Nuevo León ranks ninth highest in the country for TB incidence (out of 31 states plus the Federal District) and sixth highest for TB deaths [19]. Rates of drug-resistant TB are also excessive compared to other Mexican states [22]. Additionally, the MMA is a rapidly growing urban center with substantial variation in the socioeconomic and clinical factors that impact health.

Previous studies have documented variation in African, European, and Native American genetic ancestry in the MMA [23]–[26]. This variation is the product of intermixing between predominately Spanish men and Native American women starting around 1519, and with Africans starting in the early 16th century [26], [27]. A recent meta-analysis identified variation in associations between risk alleles and TB disease among these regional “parental” groups [16], suggesting that genetic ancestry has the potential to be informative about TB susceptibility in the MMA and other admixed populations in the Americas.

To accomplish our research objectives, we collected in-depth socioeconomic, clinical, and genetic data from 194 MMA residents with pulmonary TB disease and LTBI. The data included region-specific measures of socioeconomic status for individuals and households, clinical data related to health and lifestyle, and genetic ancestry estimated from 291,917 SNPs. We used crude association tests and multivariable logistic regression analyses to examine associations between these data and TB disease status, and we examined differences in genetic ancestry between individuals with TB disease and LTBI at SNPs located in 273 genes listed in the TB biosystem in the NCBI BioSystems database. To our knowledge, no previous studies have examined the association between genetic ancestry and TB disease in a Hispanic population in the Americas while accounting for region-specific socioeconomic and clinical factors. Our findings have broad implications for exploring the correlates of multifactorial disease in admixed groups in urban centers throughout the Americas.

## Methods

### Study design and participants

We recruited adults with pulmonary TB disease (n = 97) and LTBI (n = 97) from the Universidad Autónoma de Nuevo León (UANL) José E. González Hospital between January 2010 and February 2011. The public hospital is located in Monterrey, which is a moderate- to low-socioeconomic status municipality in the MMA [28]. The open-door policy of treating patients independent of insurance status or income attracts residents from all municipalities of the MMA. The hospital treats approximately one quarter of all new TB cases in the MMA each year [29].

TB disease status was confirmed by a positive culture. LTBI status was defined by a positive TB skin test with an induration of 10 mm or greater [30]. Among the LTBI participants, 40 were recruited from hospital clinics and waiting rooms, and 57 were recruited from hospital staff (see Table 1). The clinic-waiting room LTBI individuals were visiting the hospital for other medical reasons or were family members of patients in other clinics. The hospital staff LTBI individuals included physicians, nurses, secretaries, medical students, researchers, laboratory technicians, and custodians. LTBI individuals had no history of conversion to disease. None of the 194 participants were biologically related.

**Table 1:** Sample sizes for UANL Hospital TB disease patients and individuals with LTBI.

	TB disease patients	LTBI Total	LTBI recruited from clinics and waiting rooms	LTBI recruited from hospital staff
Full Sample	97	97	40	57
Genotyped Sample	83	59	35	24

### Ethics statement

This study was approved by the University of New Mexico (UNM) and UANL Institutional Review Boards (UNM Human Research Review Committee #09-318, UANL Ethics Committee #IN09-001). Participants gave informed written consent.

### Socioeconomic and clinical data

The socioeconomic and clinical data were collected during face-to-face interviews in private settings at the UANL Hospital. Interview questions were compiled from established Mexican national and Latin American surveys [31], [32] and TB risk assessments used by the UANL Hospital [33], [34]. The variables included 15 measures of socioeconomic status, 30 clinical variables related to health and lifestyle, three measures of indigenous ethnicity, and several demographic measures. The measures of ethnicity were based on self-report and included indigenous language capacity. Indigenous language capacity is rarely measured in disease studies; it potentially reflects recent and direct connections to Native American communities that might not be captured by self-reported ethnicity alone.

Socioeconomic variables included region-specific, household-based measures from a 10-item survey developed by the Mexican Association of Marketing Research and Public Opinion Agencies (AMAI) [31]. The survey ascertained computer and colored television ownership, type of floor, number of rooms, functioning shower, exclusive bathroom, number of lights, type of stove, number of automobiles, and education of the highest income earner in the household. The survey assigns points to each item; the points were summed to create an overall measure of socioeconomic status. We also created a three-category ordinal variable (low, medium, and high) from the point total, and nominal versions (low vs. high) of each AMAI-survey item. Clinical data were collected from interviews and medical record reviews; they included measures of tobacco use, alcohol consumption, substance abuse, disease co-morbidities, and BCG vaccination status. Individuals with extra-pulmonary TB and HIV were excluded from the study.

### Crude association tests and multivariable logistic regression

We conducted crude association tests between each variable and TB disease status to assess the unadjusted effects of socioeconomic factors, clinical measures, and genetic ancestry [18], [35]. Crude associations were assessed by Pearson's chi-square and Fisher's exact tests for categorical variables, and continuous variables were converted to Z scores and assessed with t-tests. We also used logistic regression to examine crude associations; we report odds ratios (OR) with 95% confidence intervals (CI) as measures of association. Body mass index (BMI) and body weight were excluded from analyses because weight loss is a well-known symptom of TB disease.

Before conducting these crude association tests, we examined the distribution of the social factors, clinical measures, and genetic ancestry in the LTBI sample. This examination revealed that, for many of these measures, LTBI individuals recruited from the hospital's clinics and waiting rooms differed significantly from those recruited from hospital staff (Table S2). This heterogeneity was due in part to the fact that the hospital staff included higher paid medical doctors and nurses. Based on these analyses, we determined that LTBI individuals recruited from clinics and waiting rooms were more representative of the population that was susceptible to developing TB disease.

We therefore limited multivariable logistic regression analyses to TB disease patients and clinic-waiting room LTBI individuals. We used a significance cutoff of ≤0.1 from the crude association tests for variable entry into multivariable regression, as recommended when exploring large numbers of risk factors [35]–[37]. Prior to building the multivariable model, we assessed multicollinearity among socioeconomic and clinical variables using a variance inflation factor of 2.5. TB disease was the dependent variable in the multivariable analyses, and we used both forward selection and backward elimination methods. We report OR and their 95% CI for each statistically significant variable. Statistical analyses were conducted in SAS 9.2 and PASW Statistics 18.0.

### Genetic Ancestry

The genetic data consisted of 291,917 SNPs [38] assayed from mouthwash samples. DNA was extracted using a modified Puregene extraction protocol. Extracts were genotyped at the University of Michigan's DNA Sequencing Core on the Illumina HumanCyotoSNP-12 DNA Analysis BeadChip Kit. The chip contained a subset of 2.2 million SNPs common in Yoruban, Utah Mormon, Chinese and Japanese individuals in the International HapMap Project [39]. All SNP call rates exceeded 99%. The SNPs were genotyped in a subset of 142 individuals of which 83 had TB disease and 59 had LTBI (Table 1). The TB disease subset excluded TB patients for whom we were unable to collect DNA because poor health prevented them from successfully completing the mouthwash-collection protocol. The genotyped LTBI subset was comprised of 35 individuals recruited from the hospital's clinics and waiting rooms and 24 individuals recruited from hospital staff. The SNPs were also assayed in 40 Africans, 54 Europeans, and 45 Native Americans from the CEPH-Human Genome Diversity Panel [40]; the three groups served as parental populations in the genetic ancestry analyses. The three parental groups were comprised of individuals from the following populations: Europeans: French, Adygei, Orcadian, Russian, Sardinian, and Tuscan; Native Americans: Mexican Pima, Maya, Colombian, Karitiana, and Surui; Africans: Yoruba, Mandenka, Bantu, and San.

We performed Hardy-Weinberg tests and various quality-control tests for each SNP in Plink [41]. Native American, European, and African genetic ancestry proportions were estimated for each individual using the fast, model-based approach of Alexander and colleagues [42]. We also estimated genetic ancestry at each SNP in each LTBI individual and TB disease patient using the LAMP package [43]. The analyses were performed separately for each chromosome. We used the default LAMP settings of 1E-8 for the recombination rate and 0.2 for the offset of adjacent windows. In different runs, we set the time of admixture to between 5–20 generations. The results were effectively the same for all times; we report the results for 17 generations, roughly corresponding to the midpoint between the initial arrival of Spaniards and Africans.

A fraction of the SNPs (n = 1,314) was located in 273 genes listed in the NCBI BioSystems database [44]. The database collates information from other NCBI sources about genes and proteins associated with disease and other biological systems. The database entry for TB contained all 138 of the TB candidate genes listed in the Genetic Association Database [45], an archive of the results of genetic association studies of multifactorial diseases. We used the Genetic Association Database and dbSNP [46] to identify additional potential candidate genes in areas of large difference in genetic ancestry between TB patients and LTBI individuals. We also examined SNPs located in the *FcGR1B* gene; it was recently identified in expression studies as a potentially important TB susceptibility gene [47]. We combined the results for the individual chromosomes into a single plot showing the average difference in genetic ancestry at each SNP in individuals with TB disease vs. LTBI. We considered a SNP to be potentially informative about TB-disease status if the difference in genetic ancestry between TB patients and LTBI individuals at the SNP was three standard deviations above or below the mean difference in genetic ancestry across all SNPs.

## Results

### Crude association tests and multivariable logistic regression

In the crude association tests of TB patients and LTBI recruited from clinics and waiting rooms, 12 variables were associated with TB disease status at a critical value ≤0.1 (Table S1). For the socioeconomic variables, TB disease was associated with low levels of education, a history of non-professional employment or unemployment, and lower measures for three of the individual AMAI survey items. For the clinical variables, TB disease status was positively associated with drug and alcohol abuse, smoking, diabetes, and history of incarceration. Marital status was also predictive; individuals with TB disease were less frequently married or in a civil union than LTBI individuals. Notably, self-reported indigenous ancestry, indigenous language-capacity, and genetic ancestry were not associated with TB disease. Of the 97 individuals with TB disease, 13 (13.4%) were living outside of the MMA at the time of the study; place of residence, however, was not a significant predictor of TB status.

The results of the multivariable logistic regression analyses of TB disease patients and the clinic-waiting room LTBI individuals are shown in Table 2 (N = 137). The final multivariable model revealed that marital status, diabetes, and smoking were independently associated with TB status (*p*≤0.1). The odds of being married or in a civil union were lower among TB disease patients compared to clinic-waiting room LTBI individuals (OR 0.31, 95% CI 0.14, 0.72), while the odds of having diabetes (OR 2.94, 95% CI 1.05, 8.24) and smoking (OR 1.65, 95% CI 0.91, 2.97) were greater among TB disease patients.

**Table 2:** Results of multivariable logistic regression analyses between TB disease patients and clinic-waiting room LTBI individuals.

Variable	OR	95% CI for OR Lower	95% CI for OR Upper	Significance
Marital Status*	0.311	0.135	0.715	0.006
Diabetes**	2.942	1.050	8.243	0.040
Smoking, pack-year score***	1.645	0.912	2.968	0.098

*Married, civil union (reference) vs. single, separated, divorced, widowed.

**Diabetes (reference) vs. no known diabetes.

***Total pack years calculation: (#cigarettes per day * years of smoking)/20.

As noted in the Methods section, we found that LTBI individuals recruited from clinics and waiting rooms differed significantly from LTBI recruited from hospital staff with respect to many of the socioeconomic and clinical variables. These differences are reported in Table S2. Clinic-waiting room LTBI individuals had, for example, consistently lower socioeconomic status and higher incidence of disease and substance abuse than LTBI individuals recruited from hospital staff. They also had significantly higher European genetic ancestry and significantly lower Native American genetic ancestry (Table S2).

### Genetic ancestry

**Figure 1:**
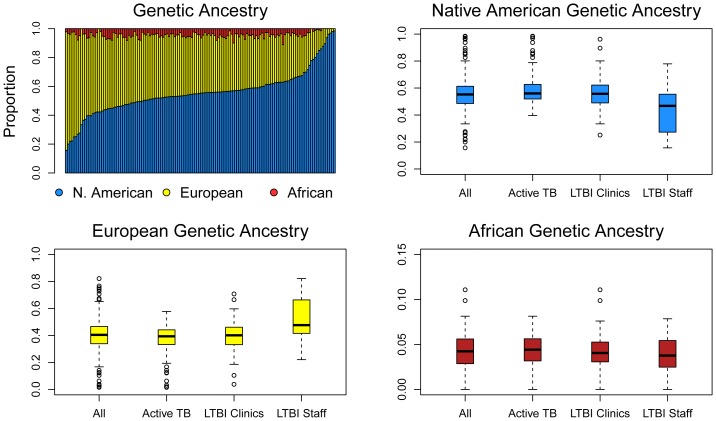
Bar plot and box and whisker plots of genome-wide genetic ancestry for 142 genotyped individuals. The bar plot shows genetic ancestry for each individuals arrayed from lowest to highest Native American genetic ancestry. Native American genetic ancestry ranged from 15.7% to 98.5%, with a mean of 55.6%. European genetic ancestry ranged from 1.5% to 82.1%, with a mean of 40.2%. African genetic ancestry ranged from 0.0% to 11.1%, with a mean of 4.2%.

The genetic ancestry estimates are plotted in Figure 1 and listed in Table 3. The top-left panel of Figure 1 shows the estimates for the full sample; these estimates fall within previously reported ranges in the MMA and northeastern Mexico by Martinez-Fierro and colleagues [48], though Cerda-Flores and colleagues reported higher European and lower Native American ancestry values [23], [24]. The standard errors of our estimates for each individual were low (mean = 0.7%), but there was substantial heterogeneity in genetic ancestry *across* individuals. The mean Native American genetic ancestry for the clinic-waiting room LTBI sample was 56.1% (s.d. = 14.0%), the mean European ancestry was 39.6% (s.d. = 13.7%), and the mean African ancestry was 4.2% (s.d. = 2.4%). The respective values for individuals with TB disease were 58.7% (s.d. = 13.2%), 37.0% (s.d. = 12.1%) and 4.3% (s.d. = 2.0%) (Table 3).

**Table 3:** Genetic ancestry. Mean proportion (standard deviation).

	TB disease patients	LTBI Total	LTBI recruited from clinics and waiting rooms	LTBI recruited from hospital staff
European	37.0 (12.1)	44.7 (15.8)	39.6 (13.7)	52.1 (16.0)
Native American	58.7 (13.2)	51.1 (15.9)	56.1 (14.0)	43.9 (16.0)
African	4.3 (2.0)	4.2 (2.2)	4.2 (2.4)	4.1 (2.0)

The remaining panels of Figure 1 show the Native American, European, and African genetic ancestry ranges for the full sample (labeled “All”), for individuals with TB disease, and for the two LTBI samples. The plots highlight the statistically significant differences in Native American and European genetic ancestry for the two LTBI samples. These differences are also documented in Table 3 (columns 3 and 4) and Table S2. Native American genetic ancestry was significantly higher in the clinic-waiting room LTBI sample, and European genetic ancestry was significantly lower. Overall, these results suggest that the MMA may be structured with respect to socioeconomic status and genetic ancestry, and that this structure may have health-related consequences.

### Genetic ancestry in genes in the TB biosystem

These analyses were restricted to 83 TB disease patients and the 35 clinic-waiting room LTBI individuals. Native American genetic ancestry ranged between 52.5% and 56.5% on the autosomes. It was relatively high on the X chromosome at 61.0%, consistent with a history of directional mating between Spanish males and Native American females. Native American genetic ancestry was higher in TB patients than in LTBI individuals on all chromosomes, and there was a corresponding deficit of European genetic ancestry.

**Figure 2:**
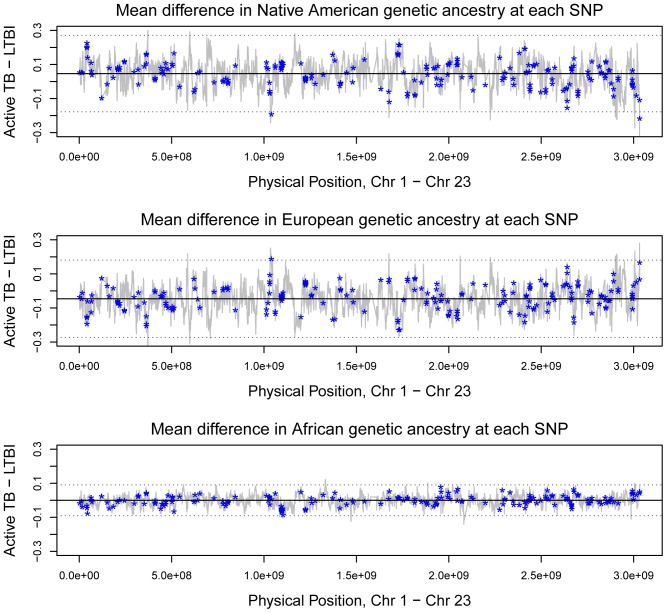
Genetic ancestry at each SNP. The y-axes in the three plots show the differences in Native American, European, and African genetic ancestry between individuals with TB disease and LTBI at each SNP. The x-axes show the physical position of each SNP, with the chromosomes plotted in order beginning with chromosome 1. The dashed lines indicate three standard deviations from the mean difference in genetic ancestry.

Figure 2 shows the results of the analyses of locus-specific genetic ancestry. It plots differences in Native American, European, and African genetic ancestry between TB patients and LTBI individuals for each SNP vs. physical position on the chromosome, beginning with position zero on chromosome 1 and terminating at the end of chromosome 23. Compared to LTBI individuals, people with TB disease had on average 4.65% greater Native American genetic ancestry (P = 2E-16, Wilcoxon signed-rank test) and 4.64% lower European genetic ancestry (P = 2E-16). African genetic ancestry did not significantly differ between the two groups (average = −9E-5).

Asterisks mark 1,314 SNPs located in 273 genes in the NCBI's TB biosystem and two SNPs in the *FcGR1B* gene. Overall, only two genes showed differences in genetic ancestry between TB disease patients and LTBI individuals at the three standard deviation level. Four SNPs in the *IL-12B* gene (Chr 5) had, on average, 3.53 standard deviations lower Native American genetic ancestry in TB disease patients than in LTBI individuals, and 3.09 standard deviations greater European genetic ancestry. Two SNPs in the *ATP6AP1* gene (Chr 23) had an average of 3.59 standard deviations lower European genetic ancestry in TB disease patients. SNPs in the surrounding regions of each gene also showed substantial differences in genetic ancestry between TB disease patients and LTBI individuals.

Twenty additional genes in the TB biosystem showed differences in genetic ancestry at the two standard deviation level. While this number is unremarkable given the fact that we examined 273 genes, many of the SNPs were located in or near regions of particularly sharp transitions in genetic ancestry, and they were linked to SNPs that also showed this sharp transition. The genes include *IL-1B* and *IL-1RN* (Chr 2), *RAB5A* (Chr 3), *NOS3* (Chr 7), *NAT2* (Chr 8), *CARD9* (Chr 9), and *NOS2A* (Chr 17).

Several other genes that have been implicated in immune system disorders (other than TB) were located in regions of large differences and sharp transitions in genetic ancestry between TB patients and LTBI individuals. These genes include *CNTN2* (Chr 1), *CBLB* (Chr 3), *PRKG1* (Chr 10), *ATXN2* (Chr 12), *SH2B3* (Chr 12) and *G6PD* (Chr 23). Previous studies [49], [50] have linked polymorphisms in two of these genes to Type 1 diabetes (*CBLB* and *SH2B3*).

## Discussion

Each of the statistically significant socioeconomic and clinical factors from our crude association tests have been identified in other studies as important determinants of the transition from LTBI to TB disease [3], [4], [6], [7], [9], [51]–[54]. Most of the socioeconomic variables are interrelated, and it is difficult to disentangle their independent effects on TB disease susceptibility, but they reflect living conditions, health-care access, and health-related lifestyle factors [8], [9], [52].

In multivariable logistic regression analyses comparing TB disease patients to clinic-waiting room LTBI individuals, we found that marital status, diabetes, and smoking were independently predictive of TB status (Table 2). Smoking is a well-known risk factor for developing TB disease [55]. Smoking impairs immune functions related to cellular defenses against TB, especially in the lungs [56]. Being married or in a lifetime partnership is increasingly recognized as a protective factor against TB disease [10], [57]. Horwitz (1971) found that being married mitigated TB disease severity and mortality, possibly due to spousal influence on treatment completion [58], [59] and the beneficial impact of “cohesive marriages” on physical and mental health [60]. Conversely, it is possible that individuals with chronic illnesses like TB may be less likely to be in a lifetime partnership due to the strain that the disease creates on relationships [60]. A recent review of Medline literature found that diabetes increased the risk or odds of TB between 1.5- and 7.8-fold [61]. A study conducted in Southern Mexico concluded that diabetes may be on par with HIV co-infection in terms of co-morbidity with TB in the country [62]. While diabetes may predispose people to TB through impaired immune function, TB may also predispose people to diabetes through impaired glucose tolerance [63].

An important finding of this study is the differences in genetic ancestry and socioeconomic status for the two LTBI groups. This result may provide evidence for assortative mating by genetic ancestry or socioeconomic status in the MMA. European ancestry is also correlated with socioeconomic status in other large urban centers in Mexico [64], [65]. A previous study of spousal choice in Hispanic populations in Mexico City and the San Francisco Bay Area showed strong correlations for assortative mating by European ancestry and Native American ancestry [66]. These results suggest that studies of the social and genetic causes of diseases in admixed population in the Americas should account for population structure.

Genetic ancestry was not predictive of the transition from LTBI to TB disease. This findings suggests one or more of the following: 1) genetic differences in TB-causing alleles do not exist between the ancestral populations that formed the Monterrey population, 2) any genetic differences that do exist contribute proportionately little to variation in TB disease compared to socioeconomic and clinical factors, 3) genome-wide genetic ancestry fails to capture locus-specific genetic differences that do exist between the parental populations, or 4) power was too low to detect existing associations.

With respect to the first two possibilities, independent of our findings, there is conflicting evidence for the existence of macrogeographic differences in the frequencies of TB-susceptibility alleles. Previous studies have identified associations between several polymorphisms in the *SLC11A1* gene and TB risk among different regions [67]–[71]. One recent meta-analysis identified statistically significant associations between four *SLC11A1* variants and TB risk in Asians and Africans, but not Europeans [16]. In contrast, a second meta-analysis in 2011 found no heterogeneity among African and European groups for TB disease risk [17]. Even if the *SLC11A1* alleles contribute to regional differences, their overall effect sizes are small; the mean odds ratio across all regions for the four *SLCA11A1* variants, for example, was only 1.29 (range 1.04–1.59) [17]. Similarly low effect sizes are reported in meta-analyses for variants of other TB-related candidate genes, such *SP110* [72], *P2X7* [73], *TIRAP* S180L [74], and a vitamin D receptor gene [13]. The measures of association are often considerably higher for socioeconomic predictors of TB. For example, in rural Mexico, drivers of TB risk include long-term indoor air pollution exposure in homes that use biomass cook stoves (OR 3.3), and households with only one room (OR 15.4) [75]. Case-control studies in other countries have reported significant odds ratios as high as 15 for social factors like ethnicity [18], and education [57]. Substance abuse also plays a major role, and a recent meta-analysis by Rehm and colleagues [76] concluded that 10% of all TB cases in the world could be attributed to alcohol.

Even though individual-level genetic ancestry was not predictive of TB status in this study, genetic ancestry differed sharply between TB disease patients and LTBI individuals at SNPs in the *IL-12B* and *ATP6AP1* genes, and at SNPs in the surrounding regions of both genes. The *IL-12B* gene encodes a subunit common to interleukin *IL-12* and *IL-23*, both of which protect against infectious diseases and cancer [77]. Variants of the gene have been linked to TB, type 1 diabetes, childhood asthma, and malaria [78]–[82]. *ATP6AP1* is located in the TB disease biosystem, but the role of polymorphisms in this gene in TB pathogenesis has not yet been specified.

There are several important limitations to this study. First, our sample sizes were relatively small. As a result, we lacked the power to identify variables with relatively small effect sizes, and to sort out the complex interactions among the socioeconomic and clinical variables. Despite these limitations, we had sufficient power to detect modest associations between TB status and genetic ancestry (in the range of 1–3%), but smaller differences in genetic ancestry may still be independently predictive of TB status in the MMA. Second, our study sample represented an urban population with access to a public hospital in a developing country, so findings might not be generalizable to rural populations, those seeking private health care, or those in developed countries. In this respect, while we attempted to examine region-specific socioeconomic factors using the AMAI survey, this effort failed to capture several factors that are likely to impact TB disease risk in Monterrey and other urban centers in developing nations. Future work would benefit, for example, from measuring community-specific measures of income inequality [9] and health service disparities [54]. Third, by restricting our locus-specific analyses to genic regions, we may have missed potential TB-risk SNPs in intergenic regions, which potentially account for variation in TB risk [12].

## Supporting Information

Table S1 **Crude association test results between TB disease patients and clinic-waiting room LTBI individuals (N = 137).**
(DOCX)

Table S2 **Crude association test results between clinic-waiting room LTBI individuals and hospital staff LTBI individuals (N = 97).**
(DOCX)

## References

[1] WHO (2011) WHO Report 2011: Global Tuberculosis Control. Geneva, Switzerland: World Health Organization.

[2] Caminero Luna JA, IUATBLD (2003) A Tuberculosis Guide for Specialist Physicians. Paris, France: International Union Against Tuberculosis and Lung Disease.

[3] Bates I, Fenton C, Gruber J, Lalloo D, Lara AM, et al (2004) Vulnerability to malaria, tuberculosis, and HIV/AIDS infection and disease. Part II: Determinants operating at environmental and institutional level. Lancet Infect Dis 4: 368–375. 15172345 10.1016/S1473-3099(04)01047-3

[4] Bates I, Fenton C, Gruber J, Lalloo D, Medina Lara A, et al (2004) Vulnerability to malaria, tuberculosis, and HIV/AIDS infection and disease. Part 1: determinants operating at individual and household level. Lancet Infect Dis 4: 267–277. 15120343 10.1016/S1473-3099(04)01002-3

[5] Cantwell MF, McKenna MT, McCray E, Onorato IM (1998) Tuberculosis and race/ethnicity in the United States: impact of socioeconomic status. Am J Respir Crit Care Med 157: 1016–1020. 9563713 10.1164/ajrccm.157.4.9704036

[6] Hargreaves JR, Boccia D, Evans CA, Adato M, Petticrew M, et al (2011) The social determinants of tuberculosis: from evidence to action. Am J Public Health 101: 654–662. 21330583 10.2105/AJPH.2010.199505 PMC3052350

[7] Ho MJ (2004) Sociocultural aspects of tuberculosis: a literature review and a case study of immigrant tuberculosis. Soc Sci Med 59: 753–762. 15177832 10.1016/j.socscimed.2003.11.033

[8] Cegielski JP, McMurray DN (2004) The relationship between malnutrition and tuberculosis: evidence from studies in humans and experimental animals. Int J Tuberc Lung Dis 8: 286–298. 15139466

[9] Harling G, Ehrlich R, Myer L (2008) The social epidemiology of tuberculosis in South Africa: a multilevel analysis. Soc Sci Med 66: 492–505. 17920743 10.1016/j.socscimed.2007.08.026

[10] Lienhardt C, Fielding K, Sillah JS, Bah B, Gustafson P, et al (2005) Investigation of the risk factors for tuberculosis: a case-control study in three countries in West Africa. Int J Epidemiol 34: 914–923. 15914505 10.1093/ije/dyi100

[11] Azad AK, Sadee W, Schlesinger LS (2012) Innate immune gene polymorphisms in tuberculosis. Infect Immun 80: 3343–3359. 22825450 10.1128/IAI.00443-12 PMC3457569

[12] Stein CM, Baker AR (2011) Tuberculosis as a complex trait: impact of genetic epidemiological study design. Mamm Genome 22: 91–99. 21104256 10.1007/s00335-010-9301-7 PMC3043369

[13] Gao L, Tao Y, Zhang L, Jin Q (2010) Vitamin D receptor genetic polymorphisms and tuberculosis: updated systematic review and meta-analysis. Int J Tuberc Lung Dis 14: 15–23. 20003690

[14] Sharma S, Rathored J, Ghosh B, Sharma SK (2010) Genetic polymorphisms in TNF genes and tuberculosis in North Indians. BMC Infect Dis 10: 165. 20537163 10.1186/1471-2334-10-165 PMC2894837

[15] Chimusa ER, Zaitlen N, Daya M, Moller M, van Helden PD, et al (2014) Genome-wide association study of ancestry-specific TB risk in the South African Coloured population. Hum Mol Genet 23: 796–809. 24057671 10.1093/hmg/ddt462 PMC3888262

[16] Li HT, Zhang TT, Zhou YQ, Huang QH, Huang J (2006) SLC11A1 (formerly NRAMP1) gene polymorphisms and tuberculosis susceptibility: a meta-analysis. Int J Tuberc Lung Dis 10: 3–12. 16466030

[17] Li X, Yang Y, Zhou F, Zhang Y, Lu H, et al (2011) SLC11A1 (NRAMP1) polymorphisms and tuberculosis susceptibility: updated systematic review and meta-analysis. PLoS One 6: e15831. 21283567 10.1371/journal.pone.0015831 PMC3026788

[18] Ladefoged K, Rendal T, Skifte T, Andersson M, Soborg B, et al (2011) Risk factors for tuberculosis in Greenland: case-control study. Int J Tuberc Lung Dis 15: 44–49. 21276295

[19] Salud SD (2011) [Situacion actual de la Tuberculosis en Mexico- Avances y Desafios]. Mexico City.

[20] WHO (2012) Tuberculosis country profiles: Epidemiology and strategy. Geneva, Switzerland: World Health Organization.

[21] CDC (2012) Reported Tuberculosis in the United States, 2011. Atlanta, GA: Centers for Disease Control and Prevention.

[22] Yang ZH, Rendon A, Flores A, Medina R, Ijaz K, et al (2001) A clinic-based molecular epidemiologic study of tuberculosis in Monterrey, Mexico. Int J Tuberc Lung Dis 5: 313–320. 11334249

[23] Cerda-Flores RM, Budowle B, Jin L, Barton SA, Deka R, et al (2002) Maximum likelihood estimates of admixture in Northeastern Mexico using 13 short tandem repeat loci. Am J Hum Biol 14: 429–439. 12112564 10.1002/ajhb.10058

[24] Cerda-Flores RM, Villalobos-Torres MC, Barrera-Saldana HA, Cortes-Prieto LM, Barajas LO, et al (2002) Genetic admixture in three Mexican Mestizo populations based on D1S80 and HLA-DQA1 loci. Am J Hum Biol 14: 257–263. 11891937 10.1002/ajhb.10020

[25] Lisker R, Ramirez E, Babinsky V (1996) Genetic structure of autochthonous populations of Meso-America: Mexico. Hum Biol 68: 395–404. 8935320

[26] Sans M (2000) Admixture studies in Latin America: from the 20th to the 21st century. Hum Biol 72: 155–177. 10721616

[27] Gorodezky C, Alaez C, Vazquez-Garcia MN, de la Rosa G, Infante E, et al (2001) The genetic structure of Mexican Mestizos of different locations: tracking back their origins through MHC genes, blood group systems, and microsatellites. Hum Immunol 62: 979–991. 11543900 10.1016/s0198-8859(01)00296-8

[28] Garza G (1996) Social and economic imbalances in the metropolitan area of Monterrey. Environment and Urbanization 8: 31–41.

[29] Molina-Torres CA, Moreno-Torres E, Ocampo-Candiani J, Rendon A, Blackwood K, et al (2010) Mycobacterium tuberculosis spoligotypes in Monterrey, Mexico. J Clin Microbiol 48: 448–455. 19940048 10.1128/JCM.01894-09 PMC2815641

[30] CDC (2011) Tuberculin Skin Testing. Atlanta: Centers for Disease Control and Prevention.

[31] AMAI (2009) Asociación Mexicana de Agencias de Investigación y Opinión Publica. Mexico D.F.

[32] ENDSA (2008) Bolivia: Encuesta nacional de demografia y salud. Instituto Nacional de Estadistica Ministerio de Salud y Deportes

[33] UCSD (2004) University of California, San Diego: Tuberculosis health assessment. San Diego, CA: UCSD.

[34] INER (2009) Instituto de Enfermedades Respiratorias. Department of Social Work, Mexican government.

[35] Brewer TF, Choi HW, Seas C, Krapp F, Zamudio C, et al (2011) Self-reported risks for multiple-drug resistance among new tuberculosis cases: implications for drug susceptibility screening and treatment. PLoS One 6: e25861. 22022459 10.1371/journal.pone.0025861 PMC3194818

[36] Hosmer DW, Lemeshow S (2000) Applied Logistic Regression, 2nd Edition. New York City, NY: John Wiley & Sons, Inc.

[37] Muture BN, Keraka MN, Kimuu PK, Kabiru EW, Ombeka VO, et al (2011) Factors associated with default from treatment among tuberculosis patients in Nairobi province, Kenya: a case control study. BMC Public Health 11: 696. 21906291 10.1186/1471-2458-11-696 PMC3224095

[38] Illumina (2012) HumanCytoSNP-12 DNA Analysis BeadChip Kit. Illimina, Inc.

[39] The International HapMap Consortium (2005) A haplotype map of the human genome. Nature 437: 1299–1320. 16255080 10.1038/nature04226 PMC1880871

[40] Cann HM, de Toma C, Cazes L, Legrand MF, Morel V, et al (2002) A human genome diversity cell line panel. Science 296: 261–262. 11954565 10.1126/science.296.5566.261b

[41] Purcell S, Neale B, Todd-Brown K, Thomas L, Ferreira MA, et al (2007) PLINK: a tool set for whole-genome association and population-based linkage analyses. Am J Hum Genet 81: 559–575. 17701901 10.1086/519795 PMC1950838

[42] Alexander DH, Novembre J, Lange K (2009) Fast model-based estimation of ancestry in unrelated individuals. Genome Res 19: 1655–1664. 19648217 10.1101/gr.094052.109 PMC2752134

[43] Sankararaman S, Sridhar S, Kimmel G, Halperin E (2008) Estimating local ancestry in admixed populations. Am J Hum Genet 82: 290–303. 18252211 10.1016/j.ajhg.2007.09.022 PMC2664993

[44] Geer LY, Marchler-Bauer A, Geer RC, Han L, He J, et al (2010) The NCBI BioSystems database. Nucleic Acids Res 38: D492–496. 19854944 10.1093/nar/gkp858 PMC2808896

[45] Becker KG, Barnes KC, Bright TJ, Wang SA (2004) The genetic association database. Nat Genet 36: 431–432. 15118671 10.1038/ng0504-431

[46] Sherry ST, Ward MH, Kholodov M, Baker J, Phan L, et al (2001) dbSNP: the NCBI database of genetic variation. Nucleic Acids Res 29: 308–311. 11125122 10.1093/nar/29.1.308 PMC29783

[47] Maertzdorf J, Repsilber D, Parida SK, Stanley K, Roberts T, et al (2011) Human gene expression profiles of susceptibility and resistance in tuberculosis. Genes Immun 12: 15–22. 20861863 10.1038/gene.2010.51

[48] Martinez-Fierro ML, Beuten J, Leach RJ, Parra EJ, Cruz-Lopez M, et al (2009) Ancestry informative markers and admixture proportions in northeastern Mexico. J Hum Genet 54: 504–509. 19680268 10.1038/jhg.2009.65

[49] Sanna S, Pitzalis M, Zoledziewska M, Zara I, Sidore C, et al (2010) Variants within the immunoregulatory CBLB gene are associated with multiple sclerosis. Nat Genet 42: 495–497. 20453840 10.1038/ng.584 PMC3786343

[50] Smyth DJ, Plagnol V, Walker NM, Cooper JD, Downes K, et al (2008) Shared and distinct genetic variants in type 1 diabetes and celiac disease. N Engl J Med 359: 2767–2777. 19073967 10.1056/NEJMoa0807917 PMC2840835

[51] de Alencar Ximenes RA, de Fatima Pessoa Militao de Albuquerque M, Souza WV, Montarroyos UR, Diniz GT, et al (2009) Is it better to be rich in a poor area or poor in a rich area? A multilevel analysis of a case-control study of social determinants of tuberculosis. Int J Epidemiol 38: 1285–1296. 19656772 10.1093/ije/dyp224 PMC2755128

[52] Lienhardt C (2001) From exposure to disease: the role of environmental factors in susceptibility to and development of tuberculosis. Epidemiol Rev 23: 288–301. 12192738 10.1093/oxfordjournals.epirev.a000807

[53] Lönnroth K, Jaramillo E, Williams B, Dye C, Raviglione M (2010) Chapter 12: Tuberculosis: the role of risk factors and social determinants. In: Blas E, Kurup AS, editors. Equity, social determinants, and public health programmes. Geneva, Switzerland: World Health Organization (WHO).

[54] Rasanathan K, Sivasankara Kurup A, Jaramillo E, Lonnroth K (2011) The social determinants of health: key to global tuberculosis control. Int J Tuberc Lung Dis 15 Suppl 2: S30–36. 10.5588/ijtld.10.0691 21740657

[55] Lin HH, Ezzati M, Murray M (2007) Tobacco smoke, indoor air pollution and tuberculosis: a systematic review and meta-analysis. PLoS Med 4: e20. 17227135 10.1371/journal.pmed.0040020 PMC1769410

[56] Ferrara G, Murray M, Winthrop K, Centis R, Sotgiu G, et al (2012) Risk factors associated with pulmonary tuberculosis: smoking, diabetes and anti-TNFalpha drugs. Curr Opin Pulm Med 18: 233–240. 22388583 10.1097/MCP.0b013e328351f9d6

[57] Tekkel M, Rahu M, Loit HM, Baburin A (2002) Risk factors for pulmonary tuberculosis in Estonia. Int J Tuberc Lung Dis 6: 887–894. 12365575

[58] Hirsch-Moverman Y, Bethel J, Colson PW, Franks J, El-Sadr W (2010) Predictors of latent tuberculosis infection treatment completion in the United States: an inner city experience. Int J Tuberc Lung Dis 14: 1104–1111. 20819254 PMC4477782

[59] Horwitz O (1971) Tuberculosis risk and marital status. Am Rev Respir Dis 104: 22–31. 5556231 10.1164/arrd.1971.104.1.22

[60] Kiecolt-Glaser JK, Newton TL (2001) Marriage and health: his and hers. Psychol Bull 127: 472–503. 11439708 10.1037/0033-2909.127.4.472

[61] Stevenson CR, Critchley JA, Forouhi NG, Roglic G, Williams BG, et al (2007) Diabetes and the risk of tuberculosis: a neglected threat to public health? Chronic Illn 3: 228–245. 18083679 10.1177/1742395307081502

[62] Ponce-De-Leon A, Garcia-Garcia Md Mde L, Garcia-Sancho MC, Gomez-Perez FJ, Valdespino-Gomez JL, et al (2004) Tuberculosis and diabetes in southern Mexico. Diabetes Care 27: 1584–1590. 15220232 10.2337/diacare.27.7.1584

[63] Young F, Wotton CJ, Critchley JA, Unwin NC, Goldacre MJ (2010) Increased risk of tuberculosis disease in people with diabetes mellitus: record-linkage study in a UK population. J Epidemiol Community Health 10.1136/jech.2010.114595 21109542

[64] Martinez-Marignac VL, Valladares A, Cameron E, Chan A, Perera A, et al (2007) Admixture in Mexico City: implications for admixture mapping of type 2 diabetes genetic risk factors. Hum Genet 120: 807–819. 17066296 10.1007/s00439-006-0273-3

[65] Torche F (2010) Educational assortative mating and economic inequality: a comparative analysis of three Latin American countries. Demography 47: 481–502. 20608107 10.1353/dem.0.0109 PMC3000020

[66] Risch N, Choudhry S, Via M, Basu A, Sebro R, et al (2009) Ancestry-related assortative mating in Latino populations. Genome Biol 10: R132. 19930545 10.1186/gb-2009-10-11-r132 PMC3091325

[67] Jin J, Sun L, Jiao W, Zhao S, Li H, et al (2009) SLC11A1 (Formerly NRAMP1) gene polymorphisms associated with pediatric tuberculosis in China. Clin Infect Dis 48: 733–738. 19193106 10.1086/597034

[68] Ates O, Dalyan L, Musellim B, Hatemi G, Turker H, et al (2009) NRAMP1 (SLC11A1) gene polymorphisms that correlate with autoimmune versus infectious disease susceptibility in tuberculosis and rheumatoid arthritis. Int J Immunogenet 36: 15–19. 19055603 10.1111/j.1744-313X.2008.00814.x

[69] Awomoyi AA, Marchant A, Howson JM, McAdam KP, Blackwell JM, et al (2002) Interleukin-10, polymorphism in SLC11A1 (formerly NRAMP1), and susceptibility to tuberculosis. J Infect Dis 186: 1808–1814. 12447767 10.1086/345920

[70] Taype CA, Castro JC, Accinelli RA, Herrera-Velit P, Shaw MA, et al (2006) Association between SLC11A1 polymorphisms and susceptibility to different clinical forms of tuberculosis in the Peruvian population. Infect Genet Evol 6: 361–367. 16461017 10.1016/j.meegid.2006.01.002

[71] Velez DR, Hulme WF, Myers JL, Stryjewski ME, Abbate E, et al (2009) Association of SLC11A1 with tuberculosis and interactions with NOS2A and TLR2 in African-Americans and Caucasians. Int J Tuberc Lung Dis 13: 1068–1076. 19723394 PMC2902362

[72] Lei X, Zhu H, Zha L, Wang Y (2012) SP110 gene polymorphisms and tuberculosis susceptibility: A systematic review and meta-analysis based on 10 624 subjects. Infect Genet Evol 10.1016/j.meegid.2012.05.011 22691368

[73] Wang X, Xiao H, Lan H, Mao C, Chen Q (2011) Lack of association between the P2X7 receptor A1513C polymorphism and susceptibility to pulmonary tuberculosis: a meta-analysis. Respirology 16: 790–795. 21470339 10.1111/j.1440-1843.2011.01976.x

[74] Miao R, Li J, Sun Z, Xu F, Shen H (2011) Meta-analysis on the association of TIRAP S180L variant and tuberculosis susceptibility. Tuberculosis (Edinb) 91: 268–272. 21419702 10.1016/j.tube.2011.01.006

[75] Garcia-Sancho MC, Garcia-Garcia L, Baez-Saldana R, Ponce-De-Leon A, Sifuentes-Osornio J, et al (2009) Indoor pollution as an occupational risk factor for tuberculosis among women: a population-based, gender oriented, case-control study in Southern Mexico. Rev Invest Clin 61: 392–398. 20184099

[76] Rehm J, Samokhvalov AV, Neuman MG, Room R, Parry C, et al (2009) The association between alcohol use, alcohol use disorders and tuberculosis (TB). A systematic review. BMC Public Health 9: 450. 19961618 10.1186/1471-2458-9-450 PMC2796667

[77] Trinchieri G, Pflanz S, Kastelein RA (2003) The IL-12 family of heterodimeric cytokines: new players in the regulation of T cell responses. Immunity 19: 641–644. 14614851 10.1016/s1074-7613(03)00296-6

[78] Davoodi-Semiromi A, Yang JJ, She JX (2002) IL-12p40 is associated with type 1 diabetes in Caucasian-American families. Diabetes 51: 2334–2336. 12086971 10.2337/diabetes.51.7.2334

[79] Morahan G, Boutlis CS, Huang D, Pain A, Saunders JR, et al (2002) A promoter polymorphism in the gene encoding interleukin-12 p40 (IL12B) is associated with mortality from cerebral malaria and with reduced nitric oxide production. Genes Immun 3: 414–418. 12424623 10.1038/sj.gene.6363909

[80] Morahan G, Huang D, Wu M, Holt BJ, White GP, et al (2002) Association of IL12B promoter polymorphism with severity of atopic and non-atopic asthma in children. Lancet 360: 455–459. 12241719 10.1016/S0140-6736(02)09676-9

[81] Morahan G, Huang D, Ymer SI, Cancilla MR, Stephen K, et al (2001) Linkage disequilibrium of a type 1 diabetes susceptibility locus with a regulatory IL12B allele. Nat Genet 27: 218–221. 11175794 10.1038/84872

[82] Morahan G, Kaur G, Singh M, Rapthap CC, Kumar N, et al (2007) Association of variants in the IL12B gene with leprosy and tuberculosis. Tissue Antigens 69 Suppl 1: 234–236. 17445208 10.1111/j.1399-0039.2006.773_3.x

