# Supplementary material for: The Effects of Socioeconomic Status, Clinical Factors, and Genetic Ancestry on Pulmonary Tuberculosis Disease in Northeastern Mexico
Source: PLoS One. 2014 Apr 11;9(4):e94303. doi: 10.1371/journal.pone.0094303 (PMC3984129; doi:10.1371/journal.pone.0094303)
Supplement: Table S1 — Crude association test results between TB disease patients and clinic-waiting room LTBI individuals (N = 137). (DOCX) [file pone.0094303.s001.docx]

**Table S1.** Crude association test results between TB disease patients and clinic-waiting room LTBI individuals (N=137)

| **Variable** | **Active TB Patients**  (n = 97)  **Count (%)** | | **Clinic-waiting room LTBI**  (n = 40)  **Count (%)** | | | **Odds Ratio**  **(95% CI)** | | ***p*-value** | |
| --- | --- | --- | --- | --- | --- | --- | --- | --- | --- |
| **Age** in years (mean ± s.d.) | 44.8 ± 16.9 | 41.4 ± 12.9 | | | 1.01 (0.99, 1.04) | | 0.20 | |  |
| **Smoking pack years*** (mean ± s.d.) | 6.7 ± 15.8 | 2.7 ± 6.1 | | | 1.03 (0.99, 1.08) | | **0.03** | |  |
| **Household crowding**** (mean ± s.d.) | 2.2 | 2.2 | | | 0.96 (0.68, 1.36) | | 0.82 | |  |
| **Current socioeconomic status***** (mean ± s.d.) | 151.2 ± 61.6 | | 164.4 ± 59.8 | | 1.0 (0.99, 1.00) | | 0.25 | |  |
| **Travel time** to UANL Hospital (minutes) (mean ± s.d.) | 67.8 ± 71.6 | | 54.0 ± 30.8 | | 1.01 (1.00, 1.01) | | 0.12 | |  |
| **European genetic ancestry** (mean ± s.d.) | 37.0 ± 12.1 | 39.6 ± 13.7 | | | 0.17 (0.01, 4.78) | | 0.36 | |  |
| **Native American genetic ancestry** (mean ± s.d.) | 58.7 ± 13.2 | 56.1 ± 14.0 | | | 4.76 (0.20, 112.1) | | 0.35 | |  |
| **African genetic ancestry** (mean ± s.d.) | 4.3 ± 2.0 | 4.2 ± 2.4 | | | 1.37 (<0.01, >99.9) | | 0.97 | |  |
| **Sex**  Female  Male | 44 (45.4)  53 (54.6) | 15 (37.5)  25 (62.5) | | | Reference  0.72 (0.34, 1.54) | | --  0.40 | |  |
| **Self-reported indigenous ethnicity and language**  Indigenous ethnicity  Non-indigenous | 18 (18.6)  79 (81.4) | 10 (25.0)  30 (75.0) | | | 0.68 (0.28, 1.65)  Reference | | 0.41  -- | |  |
| **Personal education**  Less than primary through secondary  Commercial, tech, college, specialist | 70 (72.2)  27 (27.8) | 21 (52.5)  19 (47.5) | | | Reference  0.43 (0.20, 0.91) | | --  **0.03** | |  |
| **Principal lifetime employment**  Professional, semi-professional, student  Non-professional or unemployed | 26 (26.8)  71 (73.0) | 18 (45.0)  22 (55.0) | | | Reference  2.24 (1.04, 4.82) | | --  **0.04** | |  |
| **Diabetes**  No  Yes | 68 (70.1)  29 (29.9) | 34 (85.0)  6 (15.0) | | | Reference  2.42 (0.92, 6.38) | | --  **0.07** | |  |
| **History of alcohol abuse**  No  Yes | 84 (86.6)  13 (13.4) | 39 (97.5)  1 (2.5) | | | Reference  6.04 (0.76, 47.79) | | --  **0.06** | |  |
| **Knowledge of TB airborne transmission and curable**  No  Yes | 25 (25.8)  72 (74.2) | 6 (15.0)  34 (85.0) | | | Reference  0.51 (0.19, 1.35) | | --  0.18 | |  |
| **Marijuana use**  No  Yes | 86 (88.7)  11 (11.3) | 37 (92.5)  3 (7.5) | | | Reference  1.58 (0.42, 5.98) | | --  0.50 | |  |
| **Crack/cocaine use**  No  Yes | 85 (87.6)  12 (12.4) | 40 (100.0)  0 (0.0) | | | Reference  >999.9 (<0.001, >999.9) | | --  **0.02** | |  |
| **Intravenous drug use**  No  Yes | 92 (94.9)  5 (5.2) | 40 (100.0)  0 (0.0) | | | Reference  >999.9 (<0.001, >999.9) | | --  0.14 | |  |
| **Inhalant use**  No  Yes | 93 (95.9)  4 (4.1) | 40 (100.0)  0 (0.0) | | | Reference  >999.9 (<0.001, >999.9) | | --  0.19 | |  |
| **Ever had BCG vaccination**  No, don’t know  Yes | 18 (18.6)  79 (81.4) | 5 (12.8)  35 (87.5) | | | 1.60 (0.55, 4.64)  Reference | | 0.39  -- | |  |
| **Marital status**  Single, divorced, separated, widow  Married, civil union | 50 (51.6)  47 (48.5) | 12 (30.0)  28 (70.0) | | | Reference  0.40 (0.18, 0.88) | | --  **0.02** | |  |
| **Current socioeconomic status***** (index of 10 housing, wealth, education items)  Highest, Upper-Middle  Middle  Lowest, Low-Middle | 25 (25.8)  52 (53.6)  20 (20.6) | 14 (35.0)  21 (52.5)  5 (12.5) | | | Reference  1.39 (0.61, 3.17)  2.24 (0.69, 7.28) | | --  0.44  0.18 | |  |
| **Windows in the bedroom**  No  Yes | 11 (11.3)  86 (88.7) | 0 (0)  40 (100.0) | | | | >999 (<0.001, >999)  Reference | **0.03**  -- | |  |
| **Number of rooms in house** (not including bathrooms, hallways, patios, rooftops)***  1-4  5 or more | 53 (55.2)  43 (44.5) | | | 21 (52.5)  19 (47.5) | 1.12 (0.53, 2.34)  Reference | | 0.77  -- | |  |
| **Number of complete bathrooms with shower and toilet exclusive to members of household*****  0  1 or more | 8 (8.3)  88 (91.7) | | | 1 (2.5)  39 (97.5) | 3.54 (0.43, 29.3)  Reference | | 0.24  -- | |  |
| **Presence of functioning shower in the house*****  No  Yes | 11 (11.5)  85 (88.5) | | | 1 (2.5)  39 (97.5) | 5.05 (0.63, 40.48)  Reference | | **0.09**  -- | |  |
| **Number of lights in house** (on ceiling, walls, floor lamps, desk lamps, etc.)***  0-5  6-10  11 or more | 31 (32.3)  45 (46.9)  20 (20.8) | | | 10 (25.0)  21 (15.4)  9 (22.5) | 1.40 (0.48, 4.03)  0.96 (0.38, 2.47)  Reference | | 0.54  0.94  -- | |  |
| **Material of household floor*****  Earth or cement  Other (e.g., tile) | 58 (60.4)  38 (39.6) | | | 23 (57.5)  17 (42.5) | 1.13 (0.53, 2.39)  Reference | | 0.75  -- | |  |
| **Number of cars at house** (excluding taxis)***  0  1  2 or more | 49 (51.0)  31 (32.3)  16 (16.7) | | | 19 (47.5)  14 (35.0)  7 (17.5) | 1.13 (0.40, 3.17)  0.97 (0.33, 2.88)  Reference | | 0.82  0.95  -- | |  |
| **Number of functioning color televisions in house*****  0  1  2 or more | 2 (2.1)  29 (30.2)  65 (67.7) | | | 1 (2.5)  8 (20.0)  31 (77.5) | 0.95 (0.08, 10.93)  1.73 (0.71, 4.22)  Reference | | 0.97  0.23  -- | |  |
| **Number of household computers*****  0  1 or more | 59 (62.1)  36 (37.9) | | | 17 (42.5)  23 (57.5) | 2.22 (1.05, 4.70)  Reference | | **0.04**  -- | |  |
| **Gas or electric stove in house*****  No  Yes | 2 (2.1)  94 (70.6) | | | 1 (2.5)  39 (97.5) | 0.83 (0.07, 9.42)  Reference | | 0.88  -- | |  |
| **Educational of highest income earner in household*****  Less than primary through secondary  Commercial, tech, college, specialist | 65 (69.9)  28 (30.1) | | | 20 (50.0)  20 (50.0) | Reference  0.43 (0.20, 0.92) | | --  **0.03** | |  |
| **Ever been a resident in prison**  No  Yes | 86 (88.7)  11 (11.3) | 39 (97.5)  1 (2.5) | | | Reference  5.00 (0.62, 40.00) | | --  **0.10** | |  |

* Total pack years calculation: (#cigarettes per day * years of smoking)/20

** Household crowding index: Number of people living in house / Number of rooms for sleeping; higher numbers mean more crowding, any number over 1.0 is considered crowding

*** Taken from the AMAI Mexican socioeconomic 10-item survey (2009)
