# Supplementary material for: The Effects of Socioeconomic Status, Clinical Factors, and Genetic Ancestry on Pulmonary Tuberculosis Disease in Northeastern Mexico
Source: PLoS One. 2014 Apr 11;9(4):e94303. doi: 10.1371/journal.pone.0094303 (PMC3984129; doi:10.1371/journal.pone.0094303)
Supplement: Table S2 — Crude association test results between clinic-waiting room LTBI individuals and hospital staff LTBI individuals (N = 97). (DOCX) [file pone.0094303.s002.docx]

**Table S2.** Crude association test results between clinic-waiting room LTBI individuals and hospital staff LTBI individuals (N=97)

| **Variable** | **Clinic-waiting room LTBI**  (n = 40)  **Count (%)** | **Hospital staff LTBI**  (n = 57)  **Count (%)** | **Odds Ratio**  **(95% CI)** | ***p*-value** |
| --- | --- | --- | --- | --- |
| **Age** in years (mean ± s.d.) | 41.4 ± 12.9 | 34.5 ± 12.4 | 1.04 (1.01, 1.08) | **0.01** |
| **Smoking pack years*** (mean ± s.d.) | 2.7 ± 6.1 | 0.5 ± 2.1 | 1.20 (1.0, 1.43) | **0.04** |
| **Household crowding**** (mean ± s.d.) | 2.2 ± 1.1 | 1.6 ± 0.5 | 2.64 (1.44, 4.84) | **0.002** |
| **Current socioeconomic status***** (mean ± s.d.) | 164.4 ± 59.8 | 241.5 ± 39.8 | 0.97 (0.95, 0.98) | **<0.001** |
| **Travel time** to UANL Hospital (minutes) (mean ± s.d.) | 54.0 ± 30.8 | 37.5 ± 24.5 | 1.02 (1.01, 1.04) | **0.007** |
| **European genetic ancestry** (mean ± s.d.) | 39.6 ± 13.7 | 52.1 ± 16.0 | 0.002 (<0.001, 0.18) | **0.003** |
| **Native American genetic ancestry** (mean ± s.d.) | 56.1 ± 14.0 | 43.9 ± 16.0 | 394.8 (5.1, >999.9) | **0.004** |
| **African genetic ancestry** (mean ± s.d.) | 4.2 ± 2.4 | 4.1 ± 2.0 | 51.87 (<0.001, >999.9) | 0.74 |
| **Sex**  Female  Male | 15 (37.5)  25 (62.5) | 39 (68.4)  18 (31.4) | Reference  3.61 (1.54, 8.45) | --  **0.003** |
| **Self-reported indigenous ethnicity and language**  Indigenous ethnicity  Non-indigenous | 10 (25.0)  30 (75.0) | 8 (14.0)  49 (86.0) | 2.04 (0.73, 5.75)  Reference | 0.18  -- |
| **Personal education**  Less than primary through secondary  Commercial, tech, college, specialist | 21 (52.5)  19 (47.5) | 1 (1.8)  56 (98.3) | Reference  0.012 (0.002, 0.13) | **--**  **<0.0001** |
| **Principal lifetime employment**  Professional, semi-professional, student  Non-professional or unemployed | 18 (45.0)  22 (55.0) | 56 (98.3)  1 (1.8) | Reference  68.4 (8.61, 544.1) | **--**  **<0.0001** |
| **Diabetes**  No  Yes | 34 (85.0)  6 (15.0) | 55 (96.5)  2 (3.5) | Reference  4.85 (0.93, 25.43) | **--**  **0.06** |
| **History of alcohol abuse**  No  Yes | 39 (97.5)  1 (2.5) | 57 (100.0)  0 (0.0) | Reference  >999.9 (<0.001, >999.9) | --  0.99 |
| **Knowledge of TB airborne transmission and curable**  No  Yes | 6 (15.0)  34 (85.0) | 3 (5.3)  54 (94.7) | Reference  0.32 (0.07, 1.34) | --  0.12 |
| **Marijuana use**  No  Yes | 37 (92.5)  3 (7.5) | 57 (100.0)  0 (0.0) | Reference  >999.9 (<0.001, >999.9) | --  0.98 |
| **Crack/cocaine use**  No  Yes | 40 (100.0)  0 (0.0) | 57 (100.0)  0 (0.0) | Reference  N/A | N/A |
| **Intravenous drug use**  No  Yes | 40 (100.0)  0 (0.0) | 57 (100.0)  0 (0.0) | Reference  N/A | N/A |
| **Inhalant use**  No  Yes | 40 (100.0)  0 (0.0) | 57 (100.0)  0 (0.0) | Reference  N/A | N/A |
| **Ever had BCG vaccination**  No, don’t know  Yes | 5 (12.5)  35 (87.5) | 4 (7.0)  53 (93.0) | 0.53 (0.13, 2.11)  Reference | 0.37  -- |
| **Marital status**  Single, divorced, separated, widow  Married, civil union | 12 (30.0)  28 (70.0) | 32 (56.1)  25 (43.9) | Reference  2.99 (1.27, 7.02) | --  **0.01** |
| **Current socioeconomic status***** (index of 10 housing, wealth, education items)  Highest, Upper-Middle  Middle  Lowest, Low-Middle | 14 (35.0)  21 (52.5)  5 (12.5) | 52 (91.2)  5 (8.8)  0 (0.0) | Reference  16.5 (5.0, 48.8)  >999.9 (<0.001, >999.9) | --  <0.0001  0.97 |
| **Windows in the bedroom**  No  Yes | 0 (0.0)  40 (100.0) | 3 (5.3)  54 (94.7) | >999.9 (<0.001, >999.9)  Reference | 0.98  -- |
| **Number of rooms in house** (not including bathrooms, hallways, patios, rooftops)***  1-4  5 or more | 21 (52.5)  19 (47.5) | 15 (26.3)  42 (73.7) | 0.32 (0.14, 0.76)  Reference | **0.01**  -- |
| **Number of complete bathrooms with shower and toilet exclusive to members of household*****  0  1 or more | 1 (2.5)  39 (97.5) | 0 (0.0)  57 (100.0) | <0.001 (<0.001, >999.9)  Reference | 0.99  -- |
| **Presence of functioning shower in the house*****  No  Yes | 1 (2.5)  39 (97.5) | 0 (0.0)  57 (100.0) | <0.001 (<0.001, >999.9)  Reference | 0.99  -- |
| **Number of lights in house** (on ceiling, walls, floor lamps, desk lamps, etc.)***  0-5  6-10  11 or more | 10 (25.0)  21 (52.5)  9 (22.5) | 0 (0.0)  22 (38.6)  35 (61.4) | >999.9 (<0.001, >999.9)  3.71 (1.44, 9.56)  Reference | 0.95  **0.007**  -- |
| **Material of household floor*****  Earth or cement  Other (e.g., tile) | 23 (57.5)  17 (42.5) | 14 (24.6)  43 (75.4) | 0.24 (0.10, 0.57)  Reference | **0.001**  -- |
| **Number of cars at house** (excluding taxis)***  0  1  2 or more | 19 (47.5)  14 (35.0)  7 (17.5) | 7 (12.3)  21 (36.8)  29 (50.9) | 11.24 (3.4, 37.21)  2.76 (0.95, 8.03)  Reference | **<0.0001**  **0.06**  -- |
| **Number of functioning color televisions in house*****  0  1  2 or more | 1 (2.5)  8 (20.0)  31 (77.5) | 0 (0.0)  3 (5.3)  54 (94.7) | >999.9 (<0.001, >999.9)  4.65 (1.15, 18.81)  Reference | 0.99  **0.03**  -- |
| **Number of household computers*****  0  1 or more | 17 (42.5)  23 (57.5) | 6 (10.5)  51 (89.5) | 0.16 (0.06, 0.46)  Reference | **<0.001**  -- |
| **Gas or electric stove in house*****  No  Yes | 1 (2.5)  39 (97.5) | 0 (0.0)  57 (100.0) | <0.001 (<0.001, >999.9)  Reference | 0.98  -- |
| **Educational of highest income earner in household*****  Less than primary through secondary  Commercial, tech, college, specialist | 20 (50.0)  20 (50.0) | 6 (10.5)  51 (89.5) | Reference  0.12 (0.04, 0.34) | --  **<0.0001** |
| **Ever been a resident in prison**  No  Yes | 39 (97.5)  1 (2.5) | 56 (98.3)  1 (1.8) | Reference  1.44 (0.09, 23.67) | --  0.80 |

* Total pack years calculation: (#cigarettes per day * years of smoking)/20

** Household crowding index: Number of people living in house / Number of rooms for sleeping; higher numbers mean more crowding, any number over 1.0 is considered crowding

*** Taken from the AMAI Mexican socioeconomic 10-item survey (2009)
